# Resetting the neurohormonal balance in heart failure (HF): the relevance of the natriuretic peptide (NP) system to the clinical management of patients with HF

**DOI:** 10.1007/s10741-017-9605-8

**Published:** 2017-04-05

**Authors:** Speranza Rubattu, Filippos Triposkiadis

**Affiliations:** 1 Department of Clinical and Molecular Medicine, School of Medicine and Psychology, Sapienza University of Rome, Rome, Italy; 2 Istituto di Ricovero e Cura a Carattere Scientifico (IRCCS) Neuromed, Pozzilli, Italy; 3 Department of Cardiology, Larissa University Hospital, Larissa, Greece

**Keywords:** Natriuretic peptide, Heart failure, Neurohormonal balance

## Abstract

The natriuretic peptide (NP) system, which includes atrial natriuretic peptide, B-type natriuretic peptide, and C-type natriuretic peptide, has an important role in cardiovascular homeostasis, promoting a number of physiological effects including diuresis, vasodilation, and inhibition of the renin-angiotensin-aldosterone system. Heart failure (HF) is associated with defects in NP processing and synthesis, and there is a strong relationship between NP levels and disease state. NPs are useful biomarkers in HF, and their use in diagnosis and evaluation of prognosis is well established, particularly in patients with HF with reduced ejection fraction (HFrEF). There has also been interest in their use to guide disease management and therapeutic decision making. An understanding of NPs in HF has also resulted in interest in synthetic NPs for the treatment of HF and in treatments that target neprilysin, a protease that degrades NPs. A novel drug, the angiotensin receptor neprilysin inhibitor sacubitril/valsartan (LCZ696), which simultaneously inhibits neprilysin and blocks the angiotensin II type I receptor, was shown to have a favorable efficacy and safety profile in patients with HFrEF and has been approved for use in such patients in Europe and the USA. In light of the development of treatments that target neprilysin and of recent data in relation to synthetic NPs, it is timely to review the current understanding of the role of NPs in HF and their use in diagnosis, evaluating prognosis and guiding treatment, as well as their place in HF therapy.

## Introduction

Heart failure (HF) is a complex clinical syndrome, characterized by progressive left ventricular (LV) dysfunction and impaired hemodynamics [1]. HF is associated with significant morbidity and mortality [2, 3], and novel therapies are required to improve patient outcomes.

The pathophysiology of HF is complex, involving activation of the sympathetic nervous system (SNS) and the renin-angiotensin-aldosterone system (RAAS) to maintain cardiac output and organ perfusion; however, sustained activation of these neurohormonal systems can be detrimental [1].

**Fig. 1:**
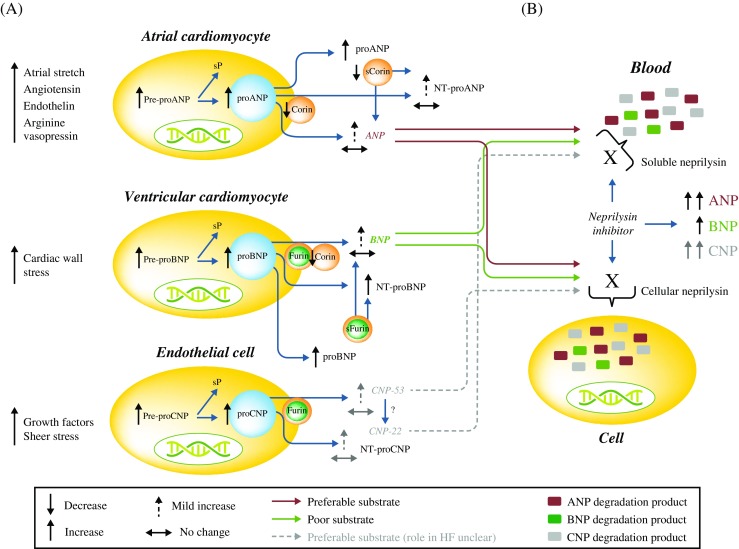
Synthesis and processing of NPs via neprilysin in patients with HF [5,6,7,8,9,10,11,12,13,14]. **a** ANP is synthesized in atrial cardiomyocytes as pre-proANP; the sP of which is cleaved to form proANP. Upon secretion, proANP is processed by membrane-bound and soluble corin, generating an N-terminal peptide (NT-proANP; 98 amino acids), and an active C-terminal peptide (ANP; 28 amino acids). In patients with HF, corin levels are decreased, resulting in an increase in predominantly unprocessed ANP. BNP is synthesized as pre-proBNP in ventricular cardiomyocytes. Removal of a sP from pre-proBNP forms proBNP, which is processed by membrane-bound and soluble furin, and corin, to release the N-terminal portion (NT-proBNP; 76 amino acids), and the biologically active C-terminal (BNP; 32 amino acids). CNP is widely expressed in the vasculature and found in high concentrations in the endothelium. CNP expression has also been reported in cardiomyocytes at gene and protein levels. CNP is synthesized as pre-proCNP; the sP of which is cleaved to form proCNP. Processing of proCNP (103 amino acids) may occur via furin to yield a53 amino acid C-terminal peptide (CNP-53), the major active form of CNP in the tissues. In the systemic circulation, a 22 amino acid form of CNP dominates (CNP-22), but the protease responsible for this cleavage is unknown. **b** Cellular and circulating soluble neprilysin are major contributors to ANP degradation. In contrast, BNP is a poor neprilysin substrate. Thus, neprilysin inhibition (e.g., via sacubitril) is most likely associated with greater augmentation of ANP activity than BNP activity. Neprilysin also has a high affinity for CNP, and as such, inhibition of neprilysin is expected to increase levels of CNP. *ANP* atrial natriuretic peptide, *BNP* B-type natriuretic peptide, *CNP* C-type natriuretic peptide, *HF* heart failure, *NP* natriuretic peptide, *NT-proANP* N-terminal proANP, *NT-proBNP* N-terminal proBNP, *Nt-proCNP* N-terminal proCNP, *sP* signal peptide, *TGF* transforming growth factor. Adapted with permission from Triposkiadis F et al. Global left atrial failure in heart failure. Eur J Heart Fail 2016;18:1307–20. © 2016 The Authors. European Journal of Heart Failure © 2016 European Society of Cardiology

Natriuretic peptides (NPs) are a family of structurally related peptides, including atrial natriuretic peptide (ANP), B-type natriuretic peptide (BNP), and C-type natriuretic peptide (CNP) [4]. They are secreted in response to increased cardiac wall stress (Fig. 1a [5–14]) to oppose the actions of the RAAS and SNS [4]. NPs mediate physiological effects including diuresis, natriuresis, vasodilation, and RAAS inhibition via natriuretic peptide receptors (NPRs) [15] and can be degraded via secretion into bodily fluids [16], through NPR-C [15] or via the protease neprilysin (which has a higher affinity for ANP and CNP than BNP (Fig. 1b) and does not degrade N-terminal [NT-]proBNP or NT-proANP) [7, 9].

Of note, defective NP processing and synthesis and resistance to bioactive NPs have been observed in HF [7, 10, 17].

In this review, we will discuss the role of the NP system in the management of HF, including the diagnostic and prognostic value of NPs, the utility of NPs in guiding therapy, and the enhancement of the NP system as a therapeutic strategy. Moreover, we will provide recommendations regarding the integration of NP measurement into HF management.

## What is the value of natriuretic peptides as biomarkers in patients with HF?

### BNP/NT-proBNP for diagnosis and prognosis

In patients with suspected HF, guidelines recommend BNP/NT-proBNP testing to confirm or exclude a diagnosis of HF [18, 19]. Levels of BNP and NT-proBNP are significantly elevated in patients with HF and increase with the severity of disease [20, 21], but are also influenced by left ventricular ejection fraction (LVEF), age, sex, renal function, sodium levels, and body mass index (BMI), as well as genetic factors and comorbidities [20, 21]. As such, the utility of BNP and NT-proBNP, while established in the diagnosis and determination of prognosis of patients with HF with reduced ejection fraction (HFrEF), may be limited in patients with HF with preserved ejection fraction (HFpEF) [22, 23]. For instance, levels of BNP and NT-proBNP are influenced by comorbidities that are frequently associated with HFpEF, with atrial fibrillation and renal disease resulting in increased levels, and obesity leading to decreased levels of these NPs [22, 24]. In addition, while BNP and NT-proBNP levels are typically lower in patients with HFpEF compared with patients with HFrEF, levels of BNP have also been reported within the normal range for patients with a preserved LVEF (in this study, diagnosis of HFpEF was based on previously published criteria, requiring a LVEF >50% and a left ventricular end-diastolic volume index <97 ml/m^2^, and was confirmed by physical exam, echocardiography, and invasive hemodynamic testing) [22, 25, 26]. These factors should be taken into consideration when interpreting NP levels in patients with suspected HFpEF.

Elevated levels of BNP and NT-proBNP are also associated with worse prognosis in terms of mortality and hospital readmission in patients with HFrEF and HFpEF, with a similar prognosis observed among patients for given values of BNP and NT-proBNP, regardless of ejection fraction [20, 22, 26, 27]. In addition, NT-proBNP values on admission for acute HF (AHF) and at discharge are predictive of all-cause mortality [28], while decreases in NT-proBNP levels during hospitalization are associated with reduced cardiovascular (CV) mortality, HF readmission [28], and all-cause mortality [29]. High and increasing NT-proBNP values have also been associated with poor outcomes [30].

### BNP/NT-proBNP levels to guide HF treatment

A BNP/NT-proBNP-guided strategy is of interest due to the underutilization of evidence-based therapies and the poor outcomes observed with current management strategies in patients with HF [31]. However, trials guided by BNP/NT-proBNP in patients with HF have generally been small and heterogeneous in design and have reported conflicting results [32]. In addition, patients with HFpEF have only been included in one of these trials (TIME-CHF; Trial of Intensified versus Standard Medical Therapy in Elderly Patients with Congestive Heart Failure) [22]. Thus, further studies are needed to clarify the potential clinical benefit of BNP/NT-proBNP-guided treatment, as well as the possible interactions with age and comorbidities [21, 33], impact on quality of life, safety and cost-effectiveness, and appropriate target cutoff values for NT-proBNP and BNP [31, 33].

Despite the above limitations, the overall impression is that BNP/NT-proBNP-guided treatment may be useful, and this is reflected in the current American College of Cardiology Foundation (ACCF)/American Heart Association (AHA) HF guidelines, which are in favor of the use of NPs to guide evidence-based treatment [19]. A prospective study (Guiding Evidence Based Therapy Using Biomarker Intensified Treatment, GUIDE-IT) to determine the impact of NT-proBNP-guided therapy on time to CV death or HF hospitalization at 12 months in ∼1100 patients with HFrEF when compared with usual care was recently terminated due to futility, with no difference in the primary outcome observed between treatment groups [34–36]. Despite this, the results are expected to provide further insight into the usefulness of biomarker-guided therapy in HF.

### BNP or NT-proBNP?

While BNP and NT-proBNP are both gold standard biomarkers for diagnosis and evaluation of prognosis in patients with HF [18, 19], NT-proBNP may be superior to BNP for the detection and evaluation of HF and the prediction of outcome [33, 37]. For instance, in the valsartan in heart failure trial, both NT-proBNP and BNP concentrations predicted all-cause mortality (hazard ratios [HRs] 2.07 and 1.87, respectively), mortality and morbidity (HRs 2.20 and 2.05, respectively), and hospitalization for HF (HRs 2.66 and 2.48, respectively) [37]. However, NT-proBNP, compared with BNP, provided significantly greater predictive value for mortality and morbidity (*p* = 0.0332) and hospitalization for HF (*p* = 0.0143), as well as marginally higher predictive value for all-cause mortality (*p* = 0.0734) when values were compared by ROC curves [37]. With a longer half-life and therefore greater circulating concentration and lower intrinsic biological variability compared with BNP [38], NT-proBNP may be a more accurate marker of ventricular stress and, consequently, determinant of prognosis in patients with HF [33]. In addition, as NT-proBNP is not degraded by neprilysin, it represents a useful biomarker for the management of patients with HF who receive therapies that inhibit this enzyme [7].

### ANP/MR-proANP for diagnosis and prognosis

In patients with HF, levels of ANP increase to a lesser extent than those of BNP (10–30-fold vs. 200–300-fold, respectively, compared with control subjects) and ANP is thought to be secreted later than BNP/NT-proBNP in response to myocardial stress [39]. The use of mid-regional proANP (MR-proANP, a more stable form of NT-proANP) in the diagnosis of AHF has been evaluated. The performance of MR-proANP in the diagnosis of AHF was slightly lower than that of proBNP, BNP, and NT-proBNP in one study [40] but, in another report, demonstrated non-inferiority to BNP and improved diagnostic accuracy in the BNP “gray zone” (BNP levels 100–500 pg/mL) and in patients with obesity [41]. In addition, MR-proANP was an independent predictor of HF diagnosis in a model that included NT-proBNP [42]. These results suggest that combined use of MR-proANP and either BNP or NT-proBNP may provide superior diagnostic accuracy than either NP alone. The current European Society of Cardiology (ESC) guidelines recommend measuring levels of BNP, NT-proBNP, or MR-proANP to exclude noncardiac causes of acute dyspnea in patients with suspected AHF [18, 19].

When interpreting ANP values in the clinic, it is important to consider them as a direct indicator of atrial size [43]. Dilated atria reflect chronicity of increased filling pressure and are a strong predictor of long-term mortality. For instance, in patients with AHF, MR-proANP had the greatest prognostic value versus BNP and NT-proBNP at 5 years [40]. In addition, MR-proANP is a strong predictor of subsequent outcome in patients with chronic HF (CHF) [44] and was the strongest predictor of CV outcome in patients with CHF followed over 15 years compared with all other considered markers [45]. Thus, MR-proANP appears to perform well in both AHF and CHF. However, as available studies are limited, the true utility of this NP for the diagnosis and prognosis of patients with HF is unknown. It is also unknown if MR-proANP may aid in guiding the management of patients with HF, as has been shown for BNP.

### CNP for diagnosis and prognosis

The role of CNP in HF has not been established. In members of the general population, CNP was reported to circulate at various concentrations, was unaffected by sex, and was weakly associated with age, but elevated levels identified a high-risk phenotype that included CV comorbidities and LV dysfunction [46]. In hospitalized patients with HF, NT-proCNP was a strong predictor of all-cause mortality and HF hospitalization at 18 months in patients with HFpEF, but not in those with HFrEF [47]. CNP has also emerged as a biomarker of structural and functional renal impairment in HF and chronic renal disease states [48].

## What strategies have been investigated to enhance the NP system for the treatment of HF?

Based on an increased understanding of the role of the NP system in HF, several therapeutic strategies to enhance the levels of NPs have been investigated. These include the administration of synthetic NPs and the inhibition of neprilysin.

### Therapeutic potential of NPs

Synthetic forms of NPs have been investigated in the treatment of HF, with some peptides being approved in specific countries for the treatment of patients with AHF [49].

Synthetic ANPs include anaritide and carperitide. Carperitide was approved in Japan for the treatment of patients with AHF in 1995, although there is limited evidence to support this indication [49, 50].

Nesiritide, a synthetic BNP, was approved for the treatment of AHF in the USA in 2001 and has been observed to improve hemodynamic parameters in patients with HFrEF [49, 50]. However, in the Acute Study of Clinical Effectiveness of Nesiritide in Decompensated Heart Failure trial including patients hospitalized with AHF (*N* = 7141), nesiritide treatment did not improve rates of rehospitalization for HF or all-cause mortality at 30 days (coprimary endpoint), had a small, nonsignificant effect on dyspnea (coprimary endpoint) and increased the rates of hypotension, compared with placebo [51]. As a result, nesiritide is not recommended for routine use in the broad population of patients with AHF [51].

Ularitide is a synthetic form of the NP urodilatin, which is secreted by the kidney. Previous studies have suggested that ularitide may have a role in the treatment of patients with AHF. In the recently completed Trial of Ularitides’s Efficacy and Safety in Patients with Acute Heart Failure in patients with AHF (*N* = 2157), ularitide treatment was associated with fewer in-hospital worsening HF events, compared with placebo [52]. No difference between treatment groups was observed for either of the two primary endpoints of CV mortality at 15 months and distribution of the hierarchical clinical composite (which characterized patients according to changes in symptoms and the occurrence of worsening HF or death within 48 h) [52, 53]. In addition, ularitide treatment did not result in improvement in the 30-day readmissions for HF, or in death from any cause or CV hospitalization at 6 months [52].

Finally, a chimeric peptide known as cenderitide, which consists of portions of CNP and *Dendroaspis* NP (a NP isolated from the venom of the eastern green mamba snake), may have beneficial renal effects, as observed in animal models [54]. Further studies are needed to investigate the potential utility of this peptide in the treatment of patients with HF, and a number of clinical studies are ongoing [54].

### Neprilysin inhibition as a therapeutic strategy

**Fig. 2:**
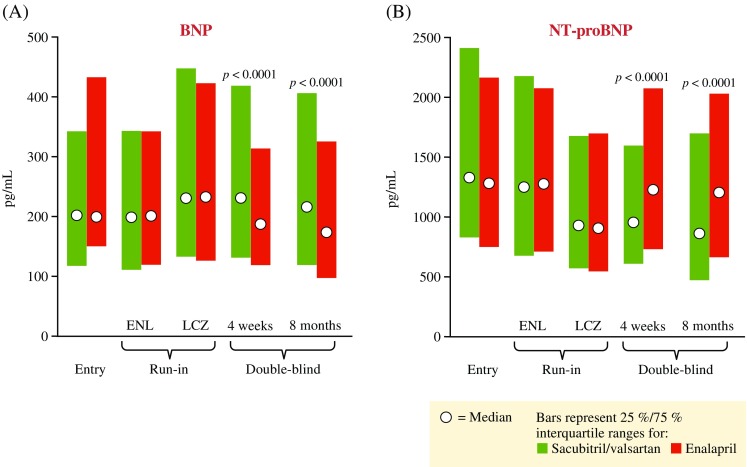
Changes in plasma levels of BNP (**a**) and NT-proBNP (**b**) following treatment with sacubitril/valsartan compared with enalapril in the PARADIGM-HF trial [55, 56]. *p* values denote significant differences between the two treatment groups. All patients received enalapril, followed by sacubitril/valsartan, during the single-blind run-in period. Groups represented here show division by final randomization group. *ACEI* angiotensin-converting-enzyme inhibitor, *ARNI* angiotensin receptor neprilysin inhibitor, *BNP* B-type natriuretic peptide, *ENL* end of the enalapril phase of the run-in period, *LCZ* end of the sacubitril/valsartan phase of the run-in period, *NT-proBNP* N-terminal pro B-type natriuretic peptide, *PARADIGM-HF* Prospective comparison of ARNI with ACEI to Determine Impact on Global Mortality and morbidity in Heart Failure trial. Reproduced with permission of Wolters Kluwer Health, Inc. Copyright © 2015, American Heart Association, Inc. From Packer M, et al. Angiotensin receptor neprilysin inhibition compared with enalapril on the risk of clinical progression in surviving patients with heart failure. Circulation 2015;131:54–61 http://circ.ahajournals.org/content/131/1/54.long

As mentioned above, defective NP synthesis/processing and NP resistance may occur in HF. In patients with HF, high concentrations of circulating soluble neprilysin have been reported and have been significantly associated with CV death, HF hospitalization, and all-cause death [5]. It should be noted that in this study, the total protein concentration of neprilysin, and not the enzymatic activity, was assessed [5]. In theory, the inhibition of neprilysin would increase the levels of bioactive forms of NPs and, therefore, enhance their beneficial effects (Fig. 2) [55, 56]. However, neprilysin has a number of physiological substrates, including angiotensin I and II, in addition to NPs [7]. Inhibition of neprilysin to enhance the beneficial effects of the NP system may also result in an increase in angiotensin II, which counteracts any potential benefit [7]. Indeed, stand-alone neprilysin inhibitors have not demonstrated efficacy beyond that of current pharmacotherapies [4]. The inhibition of neprilysin should therefore be combined with simultaneous suppression of the RAAS [7].

Combined inhibition of neprilysin, aminopeptidase (APP), and angiotensin-converting enzyme (ACE) via omapatrilat demonstrated trends towards greater efficacy when compared with enalapril [57]. However, further development of these agents was stopped due to safety concerns, primarily due to increased incidence of angioedema [4, 57]. The increased risk of angioedema with omapatrilat was thought to be due to the simultaneous inhibition of neprilysin, ACE, and APP involved in the breakdown of bradykinin, a vasoactive peptide that in turn is implicated in the pathogenesis of angioedema [7].

Sacubitril/valsartan (also known as LCZ696), an angiotensin receptor neprilysin inhibitor (ARNI), which consists of the molecular components of valsartan (an angiotensin receptor blocker, ARB) and the neprilysin inhibitor prodrug AHU377 (sacubitril), provides simultaneous inhibition of neprilysin and blockade of the angiotensin II type 1 (AT_1_) receptor (and thus, inhibition of the RAAS) [7, 58]. Sacubitril/valsartan was designed in such a way to minimize the risk of angioedema, as it only inhibits one enzyme (neprilysin) involved in the metabolism of bradykinin, with no effect on ACE [7].

Recent data indicate that the treatment approach represented by sacubitril/valsartan is beneficial. In the prospective comparison of ARNI with angiotensin-converting enzyme inhibitor (ACEI) to Determine Impact on Global Mortality and morbidity in Heart Failure (PARADIGM-HF) trial in patients with HFrEF (*n* = 8442), sacubitril/valsartan demonstrated superior efficacy when compared with ACEI enalapril on CV mortality and HF hospitalization, all-cause mortality, and quality of life as assessed by the Kansas City Cardiomyopathy Questionnaire over a mean follow-up of 27 months [59]. Treatment with sacubitril/valsartan was not accompanied by important safety concerns [59]. As such, sacubitril/valsartan was approved for the treatment of patients with HFrEF in Europe and the USA. The current ESC-HF guidelines recommend sacubitril/valsartan to replace ACEI/ARB in ambulatory patients with HFrEF who remain symptomatic despite treatment with an ACEI, beta-blocker, and MRA [18], while an ACCF/AHA guideline update recommends the use of sacubitril/valsartan in patients with chronic symptomatic HFrEF New York Heart Association class II or III who can tolerate an ACEI or ARB [60].

Sacubitril/valsartan treatment resulted in significant increases in levels of urinary cyclic guanosine monophosphate (cGMP) and BNP (Fig. 2a) compared with enalapril, reflecting inhibition of neprilysin via sacubitril and the subsequent enhancement of NP levels (via the activation of intracellular cGMP) [55]. In contrast, patients treated with sacubitril/valsartan had significantly reduced levels of NT-proBNP at 4 weeks and 8 months when compared with patients treated with enalapril, indicating reduced cardiac wall stress (Fig. 2b) [55]. A greater number of patients treated with sacubitril/valsartan attained an NT-proBNP level <1000 pg/mL when compared with enalapril [61]. An NT-proBNP level <1000 pg/mL at 1 month resulted in a 59% reduction in risk of CV death or HF hospitalization when compared with patients whose NT-proBNP levels did not decrease to <1000 pg/mL at 1 month [61]. Similarly, in the Prospective Comparison of ARNI with ARB on Management of Heart Failure with Preserved Ejection Fraction (PARAMOUNT-HF) trial in patients with HFpEF (*n* = 301), levels of NT-proBNP were significantly reduced 12 weeks following the initiation of treatment in patients who received sacubitril/valsartan compared with patients who received valsartan [56].

As NT-proBNP is not metabolized by neprilysin [7], assessment of this biomarker may accurately reflect the changes in myocardial wall stress following treatment with an ARNI [9] and, thus, aid prognosis, risk stratification, and, potentially, guidance of disease management and treatment strategy in patients with HF throughout the patient journey. This may be of particular importance in patients who appear clinically stable, but in whom disease progression, driven by neurohormonal imbalance, is ongoing despite pharmacological treatment.

The role of MR-proANP in patients with HF receiving sacubitril/valsartan therapy is currently unknown. The precursor form of ANP is not a substrate of neprilysin, and changes should mostly reflect reduced atrial filling pressure and myocardial stress during neprilysin inhibition and AT_1_ receptor blockade. Thus, plasma MR-proANP could be a valid alternative to NT-proBNP assessment. Future studies should assess the role of MR-proANP during neprilysin inhibition.

## How can the measurement of NPs be integrated into clinical practice to guide management?

NPs remain the gold standard for establishing the diagnosis and prognosis of patients with AHF and CHF. In acute cardiac care, NPs are used in a diagnostic setting to rapidly rule out AHF in patients evaluated for acute dyspnea. With cutoff values of 100 ng/L (BNP), 300 ng/L (NT-proBNP), and 120 pmol/L (MR-proANP), NPs have high negative predictive values (90–98%), but less robust positive predictive values (56–67%) [12] (although it should be noted that cutoff thresholds will vary according to the chosen assay and consequently, NP values obtained via different assays cannot be compared) [62, 63]. Moreover, NP levels may be used for disease management and treatment guidance in hospitalized patients with HF, as decreases >30% versus baseline are associated with favorable post-discharge outcomes [64].

As already discussed, several factors need to be taken into account for correct interpretation of NP levels in HF, such as age, sex, BMI, renal function, cardiac function, comorbidities, and ongoing therapies. In the latter regard, the introduction of an ARNI for the treatment of patients with HF adds a further variable. Clinicians need to know how to evaluate the clinical meaning of NP levels in patients receiving an ARNI to improve disease management. Changes in NP levels should represent the result of both the intrinsic mechanism of ARNI action and the consequent improvement of cardiac function, not excluding the impact of the abovementioned anthropometric and renal function parameters.

The interpretation of BNP testing in patients with acute dyspnea will depend on whether or not patients have received sacubitril/valsartan, although the relative increases in plasma BNP concentrations in patients treated with sacubitril/valsartan in the PARADIGM-HF trial were modest (from a median of approximately 200 ng/L at baseline to approximately 225 ng/L at 8 months) [12, 55]. It is unlikely that the diagnostic utility of BNP testing would be impaired in patients with acute dyspnea who have not been receiving sacubitril/valsartan, but using BNP levels for the guidance of treatment in patients with CHF receiving sacubitril/valsartan may not be feasible. However, as NT-proBNP is not metabolized by neprilysin [7], the interpretation of diagnostic and prognostic NT-proBNP testing in HF would likely be unaffected by previous sacubitril/valsartan treatment.

At the current stage, the precise behavior of ANP and MR-proANP in response to ARNI treatment is not known [12], and therefore, it is difficult to establish if these NPs can either mimic or even be superior to BNP/NT-proBNP assessment in patients with HF. ANP is expected to represent the most suitable marker of effective neprilysin inhibition, and experimental studies have confirmed this [58]. Higher ANP levels, despite an improvement of cardiac function, documented by both clinical and echocardiographic evaluations, should therefore be a hallmark of neprilysin inhibition. However, the upper limit of ANP should be reconsidered in HF to establish the exact level above which values are indicative of efficacious neprilysin inhibition, rather than cardiac dysfunction. On the other hand, changes in MR-proANP level may mimic those of NT-proBNP, as these peptides are not substrates of neprilysin and mostly reflect the cardiac function of treated patients. The scenario may become even more complex when considering that, in order to properly interpret the circulating NP levels in patients with HF receiving ARNI treatment, the concomitant impact of other pharmacological treatments, such as beta-blockers, MRAs, and diuretics, should be taken into account, as the overall efficacy of a HF treatment strategy certainly influences circulating levels of NPs.

Finally, in addition to determining levels of NT-proBNP, assessment of the levels of other markers that are independent from the action of ARNI, yet reflect hemodynamic changes and beneficial effects of ARNI on the CV system, such as cardiac troponins and ST2, may be valuable. It is also important to remark that both clinical and echocardiographic evaluations are necessary to integrate the information obtained with regard to NP levels, particularly in patients with symptomatic HF.

## Summary

NPs mediate beneficial physiological effects in patients with HF, including vasorelaxation and stimulation of diuresis and natriuresis, to alleviate neurohormonal and hemodynamic imbalance in HF and counteract the deleterious effects of the RAAS and SNS. Unfortunately, HF is associated with an increased ratio of unprocessed/processed NPs and, eventually, the development of NP resistance. The use of NPs in the diagnosis and evaluation of the prognosis of patients with HF is well established, particularly in patients with HFrEF. Their use in guiding the therapy of patients with HF, as well as the therapeutic potential of synthetic NPs, requires further investigation.

Morbidity and mortality remain high in patients with HF, and novel therapies are required. Through blockade of the RAAS via AT_1_ antagonism with valsartan and inhibition of neprilysin via sacubitril, sacubitril/valsartan has been shown to increase the levels of ANP and BNP (most likely the processed bioactive forms) and to decrease levels of NT-proBNP, with significant improvement in the outcomes of patients with HFrEF observed, including decreases in rates of CV mortality and HF hospitalization. Usually, increases in concentrations of ANP and BNP are suggestive of increased cardiac wall stress; however, prevention of the degradation of NPs through neprilysin inhibition (via sacubitril/valsartan) results in pharmacological elevations in the levels of these NPs. Conversely, as NT-proBNP is not degraded by neprilysin, changes in the levels of this peptide are indicative of sacubitril/valsartan-induced changes in hemodynamic profile, with decreases in NT-proBNP levels suggestive of reduced cardiac wall stress. Measurement of NT-proBNP therefore provides important information to guide optimal HF management in this setting.

ANP/MR-proANP and other biomarkers may help to interpret the clinical meaning of changes in BNP/NT-proBNP levels during neprilysin inhibition and to improve biomarker-guided disease management and, ultimately, the outcomes of patients with HF. Further studies are required to determine the clinical utility of these markers during neprilysin inhibition.
